# A Monotonic Degradation Assessment Index of Rolling Bearings Using Fuzzy Support Vector Data Description and Running Time

**DOI:** 10.3390/s120810109

**Published:** 2012-07-26

**Authors:** Zhongjie Shen, Zhengjia He, Xuefeng Chen, Chuang Sun, Zhiwen Liu

**Affiliations:** State Key Laboratory for Manufacturing System Engineering, School of Mechanical Engineering, Xi'an Jiaotong University, Xi'an 710049, China; E-Mails: zjshen.2007@stu.xjtu.edu.cn (Z.S.); chenxf@mail.xjtu.edu.cn (X.C.); sunchuang522@163.com (C.S.); Lzw1682007@126.com (Z.L.)

**Keywords:** performance degradation assessment, fuzzy support vector data description, running time, run-to-failure test, operation reliability

## Abstract

Performance degradation assessment based on condition monitoring plays an important role in ensuring reliable operation of equipment, reducing production downtime and saving maintenance costs, yet performance degradation has strong fuzziness, and the dynamic information is random and fuzzy, making it a challenge how to assess the fuzzy bearing performance degradation. This study proposes a monotonic degradation assessment index of rolling bearings using fuzzy support vector data description (FSVDD) and running time. FSVDD constructs the fuzzy-monitoring coefficient *ε̄* which is sensitive to the initial defect and stably increases as faults develop. Moreover, the parameter *ε̄* describes the accelerating relationships between the damage development and running time. However, the index *ε̄* with an oscillating trend disagrees with the irreversible damage development. The running time is introduced to form a monotonic index, namely damage severity index (DSI). DSI inherits all advantages of *ε̄* and overcomes its disadvantage. A run-to-failure test is carried out to validate the performance of the proposed method. The results show that DSI reflects the growth of the damages with running time perfectly.

## 1. Introduction

Rolling bearings, as important components of rotating machinery, not only support the load but also allow relative motion [1–4]. They are also a common failure unit due to their complex running conditions. The performance of bearings directly affects the operation reliability of the whole equipment [5–9], therefore, fault diagnosis and degradation assessment based on condition monitoring have been a key means to ensure the reliable operation of equipment, reduce the production downtime and save maintenance costs, *etc.* [10].

Vibration analysis is a powerful tool for fault diagnosis and degradation assessment [11–14]. The performance of rolling bearings is influenced by many factors, such as rotation speed, temperature, and lubrication conditions [15,16]. The running state is the final results of those factors. The vibrations reflect synthetically the present running state of the bearing. Many researches have been developed about the feature extraction from the vibration signals to assess the performance degradation. Time-domain features and frequency-domain features are the first choice because of the easy calculation and the definite physical meaning. Root mean square (RMS), Kurtosis Factor, the average amplitude of the defective frequency and its first six harmonics were successively used in [17–19], but the original features have different advantages and limitations as well. For example, RMS is a good stability feature which steadily grows with the fault development of the bearing. However, it is difficult to discover the incipient defects by RMS. On the contrary, Kurtosis Factor is sensitive to impulse faults. It distinctly appears at the initial fault stage while it decreases with the fault development. Kurtosis Factor has high sensitivity for incipient faults, but the poor stability for the serious damage. Few original features satisfy the conditions of sensitivity and stability simultaneously. A composite index is necessary which is both sensitive to the initial defect and rises stably as the damage grows.

One challenge is how to structure an intelligent assessment model based on the original features. Several scholars have proposed some comprehensive indexes and obtained impressive results. Qiu *et al.* proposed the minimum quantization error (MQE) for the assessment of the performance degradation of rolling bearings based on Self Organizing Map (SOM) and optimal wavelet filters [20]. Huang *et al.* applied MQE to the residual life for ball bearings using a back propagation neural network [21]. Pan *et al.* developed three indicators spectral entropy, health index and degradation indicator using information entropy, wavelet packet-support vector data description and lifting wavelet packet decomposition-fussy c-means, respectively [22–24]. Yu utilized a locality preserving projection for feature extraction, and provided the two complementary indexes, exponential weighted moving average (EWMA) and negative log likelihood probability–based EWMA statistic (NLLPEWMA) to assess the bearing performance degradation [25,26]. Caesarendra *et al.* proposed the combination of relevance vector machine (RVM) and logistic regression (LR) for the performance degradation assessment and prediction from incipient defects to final failure [27,28]. These indicators reflect the degradation trends of bearing performance to different degrees. However, sometimes a monotonic index is necessary to reflect the damage development more accurately. Little research about monotonic indexes is reported. The monotonic index should be constructed for two reasons. Firstly, the damage is irreversibly growing with the operation time. If the assessment index is oscillating, it is unable to reflect the damage development trends perfectly. Secondly, the damage shows accelerated growth with the running time. Few indicators reflect these accelerated relationships.

This study proposes a monotonic degradation assessment index for rolling bearings using fuzzy support vector data description (FSVDD) and running time. FSVDD is the combination of fuzzy mathematics theory and SVDD, and deals well with the fuzzy matter in small samples. The performance degradation of bearings belongs to this situation. The performance degradation with the strong fuzziness is the intermediate process between the normal running and the final failure. The initial defect, the final failure and the degrees of damage severity with different moments are hard to identify. Meanwhile, the dynamic information which reflects the change of bearing states is fuzzy and random. Some deviations appear when the fuzzy matter is dealt with by the deterministic mathematic method. Besides, the bearing monitored during its whole life is rare due to the difficulties in engineering. Support vector data description (SVDD) is an excellent method of one-class classification for small samples, with the advantages of robustness and high computation [29,30]. However, SVDD without the fuzzy identification function is unable to discriminate the damage severity degrees of samples. FSVDD is the fuzzy computing method based on SVDD. It introduces the fuzzy membership degree into the kernel function [31,32]. The fuzzy membership degree indicates the importance according to the damage severity degree. FSVDD constructs the fuzzy-monitoring membership which describes the accelerating relationships between the damages and running time. In addition, the running time is used to construct the monotonous growth of the bearing damage development.

This paper is organized as follows: In Section 2, the basic theory of FSVDD is introduced, and Section 3 presents the performance assessment method based on FSVDD and running time. The bearing run-to-failure tests and the related analysis are provided in Section 4. Section 5 provides the conclusions from the above studies.

## 2. Fuzzy Support Vector Data Description

### 2.1. Support Vector Data Description

**Figure 1.**
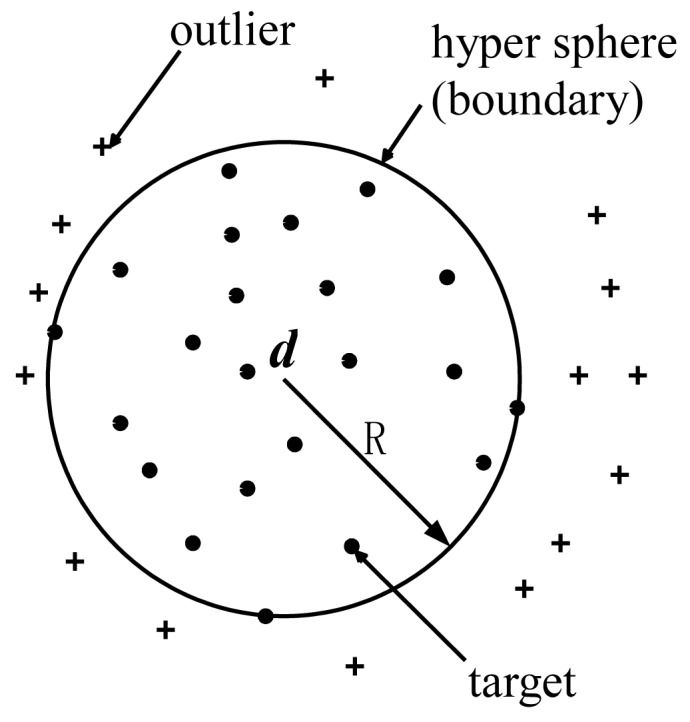
Schematic diagram of support vector data description.

Support vector data description proposed by Tax and Duin [29] is an excellent method of one-class classification. It is inspired by the idea of the support vector machine machines put forth by Vapnik [33]. The basis idea of SVDD is to find a spherically shaped decision boundary with minimum volume containing all (or most) targets, as described in Figure 1.

Assuming a training set contains *n* vectors of objects {*x_i_, i* = 1,2,…,*n*}. We try to find the minimum-volume hypersphere which contains all or most normal objects. This hypersphere is described by center *d* and radius *R*, and satisfies the following function:

(1) $$\begin{array}{l} \min L(R,d,\xi )=R^{2}+C\sum_{i=1}^{n}\xi_{i},\\ \text{s.t.}\;\{\begin{array}{c} {\left(x_{i}-d\right)}^{T}(x_{i}-d)\leq R^{2}+\xi_{i},\\ \xi \geq 0,i=1,2,\cdots ,n \end{array} \end{array}$$

where *ξ_i_* is slack variable to enhance the robustness and *C* is a penalty constant which gives the trade-off between volume of the hypersphere and errors. Then constraint condition is incorporated into the objective function by Lagrange multipliers:

(2) $$L(R,d,\alpha_{i},\xi_{i},\gamma_{i})=R^{2}+C\sum_{i=1}^{n}\xi_{i}-\sum_{i=1}^{n}\alpha_{i}\left[R^{2}+\xi^{2}-\left(x_{i}^{2}-2dx_{i}+d^{2}\right)\right]-\sum_{i=1}^{n}\gamma_{i}\xi_{i}$$

where *α_i_* ≥ 0, *γ_i_* ≥ 0 are the Lagrange multipliers. *L* should be minimized with respecting to *R, d, ξ_i_*. Taking the partial derivatives of *L* and *R, d, ξ_i_* to zero, new constraints are given as follows:

(3) $$\begin{array}{ccc} \sum_{i=1}^{n}\alpha_{i}=1, & d=\sum_{i=1}^{n}\alpha_{i}x_{i}, & \gamma_{i}=C-\alpha_{i} \end{array}$$

Equation (3) deduces:

(4) $$0\leq \alpha_{i}\leq C$$

Reconstituting Equation (2) by Equation (3) results in:

(5) $$\begin{array}{l} \max L(\alpha )=\sum_{i=1}^{n}\alpha_{i}(x_{i}\cdot x_{i})-\sum_{i=1,j=1}^{n}\alpha_{i}\alpha_{j}(x_{i}\cdot x_{j})\\ \text{s.t.}\;\{\begin{array}{c} \sum_{i=1}^{n}\alpha_{i}=1,\\ 0\leq \alpha_{i}\leq C \end{array} \end{array}$$

All *α_i_* are obtained by solving Equation (5) and only a small set of them are not zero. The objects with *α_i_* > 0 are the support vectors. The radius *R* is acquired by any support vector *x_k_*:

(6) $$R^{2}=(x_{k}\cdot x_{k})-2\sum_{i=1}^{n}\alpha_{i}(x_{i}\cdot x_{k})+\sum_{i,j=1}^{n}\alpha_{i}\alpha_{j}(x\cdot x_{j})$$

For a new object *z*, its distance to the center *d* is described as follows:

(7) $$R_{z}^{2}={\left\|z-d\right\|}^{2}=(z\cdot z)-2\sum_{i=1}^{n}\alpha_{i}(z\cdot x_{i})+\sum_{i,j=1}^{n}\alpha_{i}\alpha_{j}(x_{i}\cdot x_{j})$$

when the data in the input space are not linearly predicted, a non-linear vector function *ϕ*(*x*) is needed to map them onto a high-dimensional feature space. Consequently, the kernel function, *K*(*x_i_,x_j_*) = *ϕ*(*x_i_*)*ϕ*(*x_φ_*), is brought in SVM which maps the original data points to a high-dimensional feature space and transforms the nonlinear problem to a linear model. When the kernel function is introduced, Equations (5)–(7) are transformed to the following form:

(8) $$\begin{array}{l} \max L(\alpha )=\sum_{i=1}^{n}\alpha_{i}K(x_{i}\cdot x_{i})-\sum_{i=1,j=1}^{n}\alpha_{i}\alpha_{j}K(x_{i}\cdot x_{j})\\ \text{s.t.}\;\{\begin{array}{c} \sum_{i=1}^{n}\alpha_{i}=1,\\ 0\leq \alpha_{i}\leq C \end{array} \end{array}$$

(9) $$R^{2}=K(x_{k}\cdot x_{k})-2\sum_{i=1}^{n}\alpha_{i}(x_{i}\cdot x_{k})+\sum_{i,j=1}^{n}\alpha_{i}\alpha_{j}K(x\cdot x_{j})$$

(10) $$R_{z}^{2}={\left\|z-d\right\|}^{2}=K(z\cdot z)-2\sum_{i=1}^{n}\alpha_{i}K(z\cdot x_{i})+\sum_{i,j=1}^{n}\alpha_{i}\alpha_{j}(x_{i}\cdot x_{j})$$

Then the monitoring coefficient of new object *z* to the hypersphere boundary is given by:

(11) $$\varepsilon =(R_{z}-R)/R$$

If *ε* ≤ 0, *z* is target, or else it is an outlier.

### 2.2. Fuzzy Support Vector Data Description

SVDD only simply identifies the normal samples and the fault samples, but is unable to accurately distinguish the samples of different degrees of damage severity. Then FSVDD is generated by introducing the fuzzy mathematics theory into SVDD to describe the development process from the initial defect to final failure [30–32].

A fuzzy membership degree *s_i_*, 0 ≤ *s_i_* ≤ 1, is introduced with each training sample *x_i_*, which is regarded as the importance according to the damage severity degree. The bigger the fuzzy membership degree *s_i_* is, the more important the training sample *x_i_* becomes. Suppose we are given a training set *S* of labeled training samples with the fuzzy membership degrees:

$$S=\{(x_{1},s_{1}),(x_{2},s_{2}),\cdots ,(x_{n},s_{n})\}$$

The fuzzy non-linear vector function is redefined as *ϕ̄*(*x, s*)=(*s* + 1)*ϕ*(*x*), and kernel function is also recounted as Equation (12):

(12) $$\bar{K}(x_{i},x_{j})=(s_{i}+1)\phi (x_{i})\cdot (s_{j}+1)\phi (x_{j})=(s_{i}+1)(s_{j}+1)K(x_{i}\cdot x_{j})$$

Equations (8)–(11) are transformed to the following form:

(13) $$\begin{array}{l} \max L(\alpha )=\sum_{i=1}^{n}\alpha_{i}{\left(s_{i}+1\right)}^{2}K(x_{i}\cdot x_{i})-\sum_{i=1,j=1}^{n}\alpha_{i}\alpha_{j}(s_{i}+1)(s_{j}+1)K(x_{i}\cdot x_{j})\\ \text{s.t.}\;\{\begin{array}{c} \sum_{i=1}^{n}\alpha_{i}=1,\\ 0\leq \alpha_{i}\leq C \end{array} \end{array}$$

(14) $${\bar{R}}^{2}={\left(s_{k}+1\right)}^{2}K(x_{k}\cdot x_{k})-2(s_{k}+1)\sum_{i=1}^{n}\alpha_{i}(s_{i}+1)K(x_{i}\cdot x_{k})+\sum_{i,j=1}^{n}\alpha_{i}\alpha_{j}(s_{i}+1)(s_{j}+1)K(x_{i}\cdot x_{j})$$

(15) $${\bar{R}}^{2}={\left(s+1\right)}^{2}K(z\cdot z)-2(s+1)\sum_{i=1}^{n}\alpha_{i}(s_{i}+1)K(z\cdot x_{i})+\sum_{i,j=1}^{n}\alpha_{i}\alpha_{j}(s_{i}+1)(s_{j}+1)K(x_{i}\cdot x_{j})$$

(16) $$\bar{\varepsilon }=({\bar{R}}_{z}-\bar{R})/\bar{R}$$

where *ε̄* is the fuzzy-monitoring coefficient.

From the comparison of Equations (8)–(11) and Equations (13)–(15), we find that they are same when *s_i_* = 0. Hence, SVDD could be seen as a special type of FSVDD.

## 3. Degradation Assessment Method Based on FSVDD and Running Time

SVM, SVDD and FSVDD are excellent classifiers for small samples, and have been widely applied to fault diagnosis [30–32,34,35]. However, few studies have reported the performance degradation assessment based on them, because the degradation assessment is different from fault diagnosis. The purpose of fault diagnosis is to identify the bearing faults, while the performance degradation assessment is to find an index which truly reflects the damage growth process. The performance degradation assessment is the development and the extension of fault diagnosis. In this study, FSVDD is applied to construct a degradation assessment indicator with the running time.

The bearing damage undergoes sustained development with the running time after the initial defect. The damage development trend should be an increasing function of time. Hence we consider that the running time is used to construct a degradation assessment indicator which could reflect the damage development of the bearing more accurately. On the other hand, the vibration signal of bearings usually contains lots of random information which may cause some deviations between the signal and the true damage. It is able to reduce the influence of the vibration randomness that the running time is introduced into the construction of the assessment indicator.

The performance degradation of the bearing is a continuous process of change. A new bearing is installed into the rotating shaft. After a short running-in, it enters a long-term stable working period. Then the minor fault appears, and the defect gradually increases as the bearing fault develops. Finally the bearing fails with a serious defect. According to the running process, the bearing life is divided into three parts: normal stage, degeneration stage and failure stage. In the beginning of the normal stage, there may be a short run-in period. The degradation assessment model is provided based FSVDD and running time as follows:

### Step 1: Feature extraction and selection

The time-domain features of vibration signals are extracted. The stability features, such as RMS, square-root amplitude (SRA) and absolute average values (AAV), and the sensitive feature as Kurtosis factor are selected as the inputs of FSVDD in the meantime.

### Step 2: Training and testing based on FSVDD

The function of fuzzy membership degree *s_i_* associated with each sample *x_i_* is given by a rising ridge distribution such as Equation (17) due to the rising statistic features, the fuzzy bounds of degradation beginning and final failure:

(17) $$s_{i}(x_{i})=\{\begin{array}{ll} 0, & 0\leq E(x_{i})<a_{1}\\ \frac{1}{2}+\frac{1}{2}\sin\frac{\pi }{a_{2}-a_{1}}\left(E(x_{i})-\frac{a_{1}+a_{2}}{2}\right), & E(x_{i})\in [a_{1},a_{2}]\\ 1, & E(x_{i})>a_{1} \end{array}$$

where *E*(*x_i_*) is the energy of *x_i_, a*_1_ is the maximum energy of normal state and *a_2_* is the minimum energy of failure state.

The normal samples are trained to construct the hypersphere. Then the fuzzy-monitoring coefficient *ε̄* for each testing sample *z* is obtained by testing all samples. The fuzzy matter, degradation assessment, is made clear by the fuzzy-monitoring coefficient *ε̄*.

### Step 3: Degradation assessment

A monotonic degradation assessment index of rolling bearing, damage severity index (DSI), is described based on FSVDD and running time as follows:

(18) $$DSI_{i}=\{\begin{array}{ll} 0, & t_{i}<t_{id}\\ \max(\bar{\varepsilon_{i}},\frac{t_{i}}{t_{i-1}}DSI_{i-1}), & t_{i}\geq t_{id} \end{array}$$

where *i* is the sample number, *t_i_* is its corresponding running time, *t_id_* is the initial defect time and *DSI_i_* is the damage severity index of the present moment.

Two assumptions are necessary for Equation (18). Firstly, the bearing has already used up the run-in period, and moved into the latter stage. Secondly, the bearing runs continuously and has a steady working condition, that is, the fuzzy-monitoring coefficient *ε̄* does not have wild points. The performance degradation begins at the initial defect time, *t_id_*, which is found by the constraint *ε̄_i_* > 0. The non-equality *ε̄_i_* > 0 means that its corresponding sample is an outlier. If the bearing is still in the run-in period, the fuzzy-monitoring coefficient *ε̄_i_* may be more than zero, but the corresponding sample is not the initial defect. After the run-in period, one point corresponds to *ε̄_i_* > 0, but its neighbor points are all *ε̄_i_* < 0. The point is taken for the wild point. Only when the points with *ε̄_i_* > 0 continuously appear three times, the first point among them is considered as the initial defect time. After the initial defect, the wild points of the parameter *ε̄* should also be rejected. The deletion of the wild point assures that DSI gets rid of the influence of the vibration randomness. If the amplification of one point is more than 150% compared with the amplitude of former point except the initial defect, the point is considered as the wild point and its amplitude should be replaced by the mean amplitude of the neighbor points.

In Equation (18), the fuzzy-monitoring coefficient *ε̄* and the running time are used to construct the novel indicator DSI which has at least two meanings. On the one hand, *DSI_i_* = *ε̄_i_* when *ε̄_i_* is bigger, which means the emergence of new fault. On the other hand, the defect of the current moment is not less than that of the former moment, even if no new defect appears, because the damage development is irreversible. The running time makes *DSI* increase slowly when the running state of the bearing is balanced. DSI inherits the defuzzification ability as good as the fuzzy-monitoring coefficient *ε̄*, and means the quantitative measurement of the fault severity degree. A higher DSI value indicates more serious faults.

## 4. Experiment and Simulation Verification

### 4.1. Bearing Run-to-Failure Test

**Figure 2.**
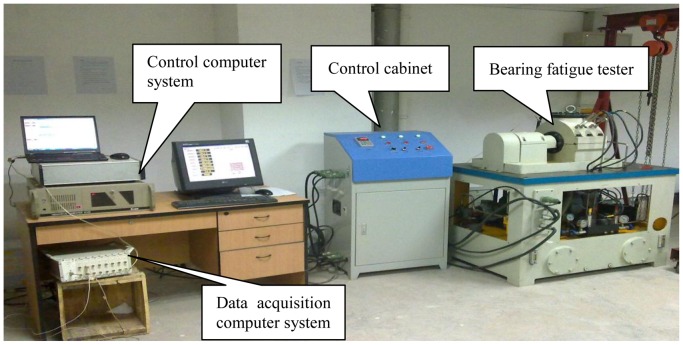
Test bench for bearing run-to-failure.

**Figure 3.**
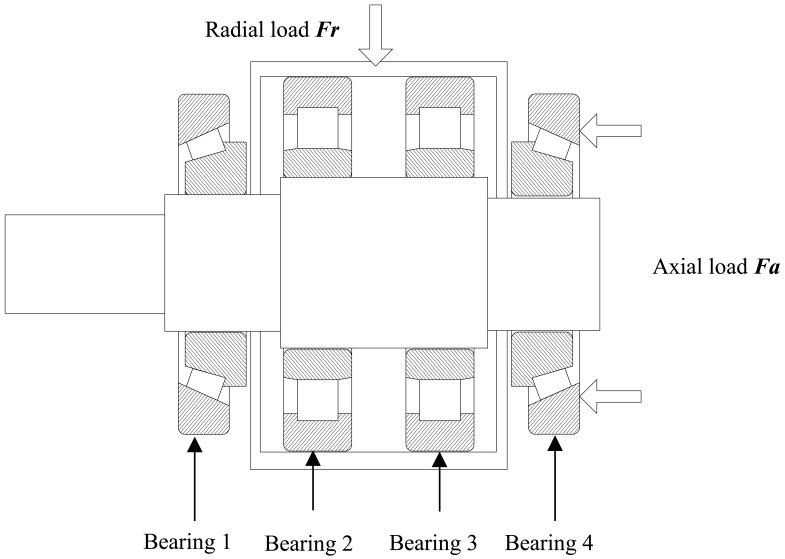
Load diagram of four bearings.

The bearing run-to-failure test is carried out under constant load conditions on the bearing tester to reflect the defect propagation processes. The test bench shown in Figure 2 is made up of power and drive system, main body, hydraulic loading system, lubrication system, control system and an independent data acquisition system, *etc.* It is designed as a simply supported beam structure, as described in Figure 3.

**Figure 4.**
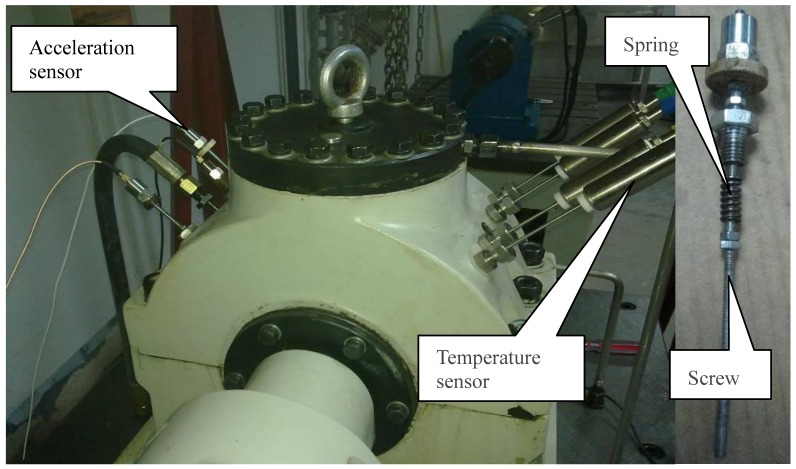
Location of accelerometer sensors and temperature sensors.

The two test bearings, 30311 tapered roller bearings, are installed on both ends of the shaft, while the two steady bearings, N312 cylindrical roller bearings, are fixed at the middle of the shaft. The axial load *F_a_* is directly inflicted on bearing 4, and transmitted to bearing 1 by the shaft. The radial load *F_r_* is exerted on the steady bearings, transferred to the shaft, and finally inflicted on the test bearings. Table 1 lists the parameters of test bearings and steady bearings. The vibration signals are acquired with a sampling frequency of 10 kHz per channel by YE6267 dynamic data collection and analysis system by Sinocera Piezotronics Inc. The data recorder is equipped with low-pass filters at the input stage for anti-aliasing. Each sample with 32,768 points is collected every five min. The acceleration sensor, as shown in Figure 4, is the Lance LC0401 High Sensitivity ICP accelerometer.

The vibration signals are transmitted by the screw which touches the outer-race of bearing. The spring exerts a pre-tightening force to ensure the contact of the screw and the outer-race. The acceleration sensor is fixed on the screw with the insulation spacer that insulates electromagnetic interference. In addition, the temperatures of four bearings are monitored by the thermocouple sensors. The axial load *F_a_* and the radial load *F_r_* of single bearing are 15 kN and 27 kN, respectively. The rotation speed is kept constant at 1,500 r/min. The characteristic frequencies are calculated by Equations (19)–(22):

(19) $$f_{c}=\frac{f_{r}}{2}\left(1-\frac{d}{D_{P}}\cos\theta \right)$$

(20) $$f_{o}=\frac{n}{2}\left(1-\frac{d}{D_{P}}\cos\theta \right)\;f_{r}$$

(21) $$f_{i}=\frac{n}{2}\left(1+\frac{d}{D_{P}}\cos\theta \right)\;f_{r}$$

(22) $$f_{e}=\frac{D_{P}}{2d}\left[1-{\left(\frac{d}{D_{P}}\right)}^{2}\cos^{2}\theta \right]\;f_{r}$$

where *f_r_, f_c_, f_o_, f_i_, f_e_* are the shaft frequency, the cage defect frequency, the outer-race defect frequency, the inner-race defect frequency and the rolling element defect frequency, respectively. *d, D_p_*, are the rolling element diameter and pitch diameter. *n* is the number of rolling elements and *θ* is the contact angle. Besides, the ball spin frequency is *f_b_* = *f_e_*. Five characteristic frequencies are shown in Table 2.

### 4.2. Results and Discussion

There are three successful experiments in the present study. In each test, there is only one failure bearing which has huge vibration signals overwhelming those of the other three normal bearings. It may result from the individual factors of each bearing. The test 1 failure bearing has inner-race defects while the tests 2 and 3 failure bearings exhibit rolling element defects.

**Figure 5.**
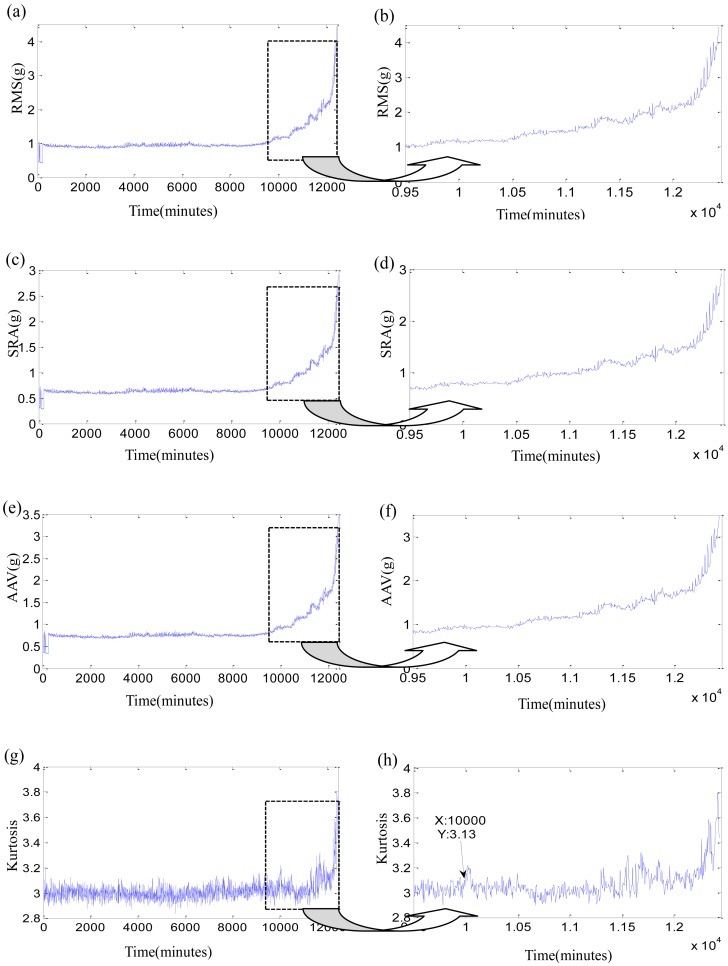
Four time-domain features of test 1 failure bearing: (**a**) RMS during the whole life; (**b**) local enlargement of RMS; (**c**) SRA during the whole life; (**d**) local enlargement of SRA; (**e**) AAV during the whole life; (**f**) local enlargement of AAV; (**g**) Kurtosis factor during the whole life; (**h**) local enlargement of Kurtosis factor.

**Figure 6.**
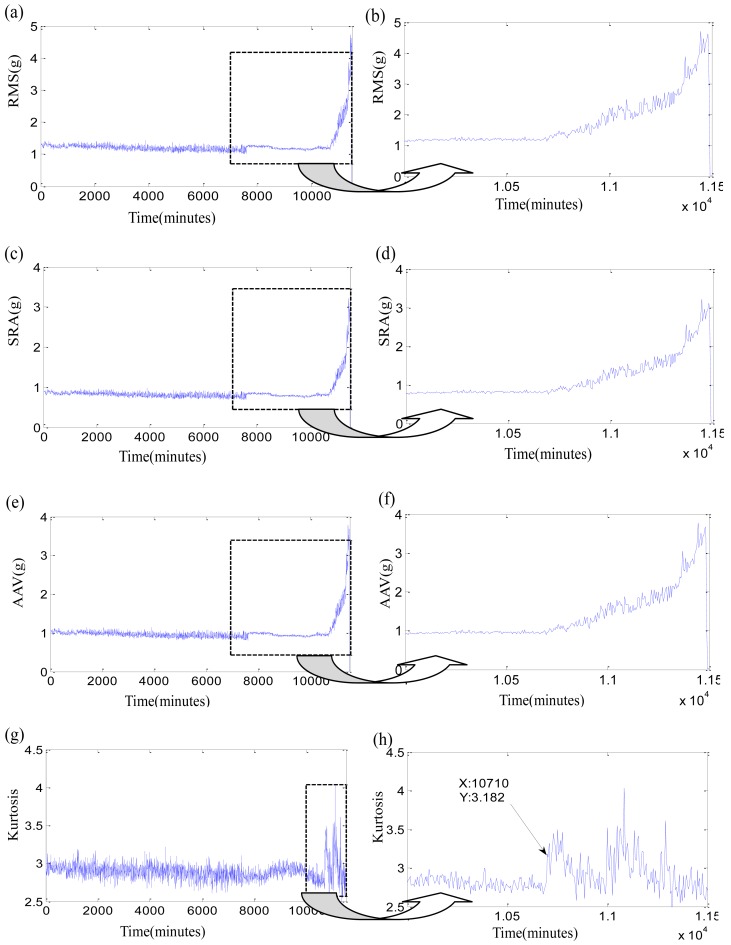
Four time-domain features of test 2 failure bearing: (**a**) RMS during the whole life; (**b**) local enlargement of RMS; (**c**) SRA during the whole life; (**d**) local enlargement of SRA; (**e**) AAV during the whole life; (**f**) local enlargement of AAV; (**g**) Kurtosis factor during the whole life; (**h**) local enlargement of Kurtosis factor.

**Figure 7.**
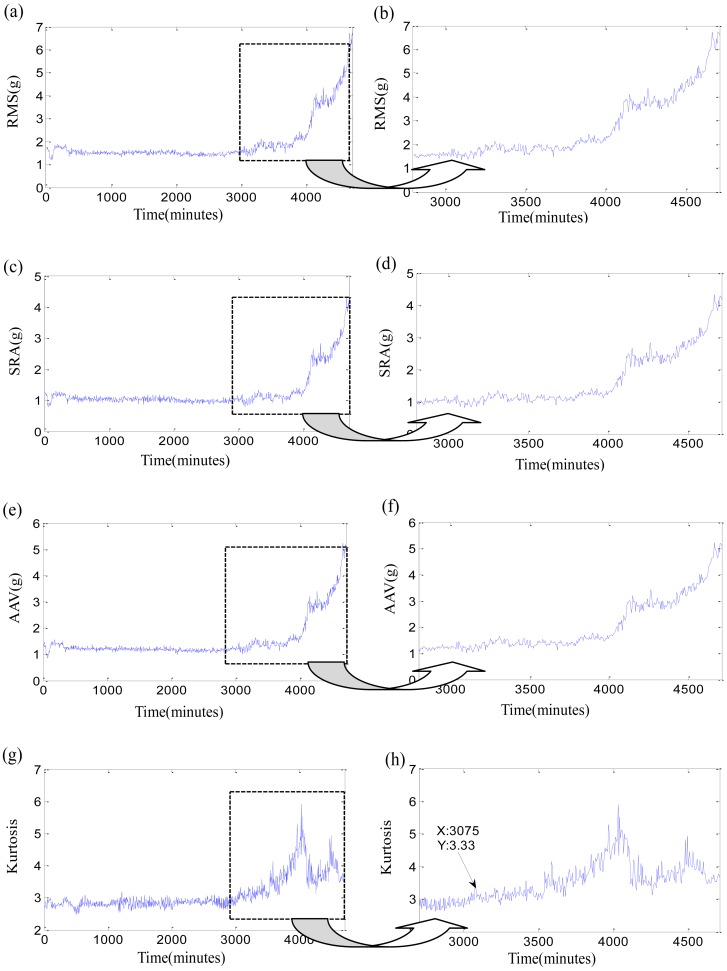
Four time-domain features of test 3 failure bearing: (**a**) RMS during the whole life; (**b**) local enlargement of RMS; (**c**) SRA during the whole life; (**d**) local enlargement of SRA; (**e**) AAV during the whole life; (**f**) local enlargement of AAV; (**g**) Kurtosis factor during the whole life; (**h**) local enlargement of Kurtosis factor.

Firstly, the original time-domain features are researched. Three stability features, such as RMS, square-root amplitude (SRA), absolute average values (AAV), and one sensitive feature as Kurtosis factor of three bearings are listed in Figures 5–7. In each Figure, the right subgraph is the local enlargement of the left subgraph to describe the variance of each feature in degradation period more distinctly. These figures at least tell us the following: (1) The normal periods usually are obviously longer than the degradation period which is verified in reference [36]. (2) In the normal period, the three stability features have placid trends while they grow continuously with the development of faults in the degradation period, but there is not an obvious impulse for each stability feature when the initial defect occurs. Three features of test 2 failure bearing have large changes at 7,600 min. It could be resulted from a dismounting and reinstallation. (3) Kurtosis factor is bumping up when the incipient fault appears, but its subsequent behaviors are bad. Kurtosis factor could be used to roughly discriminate the initial defect times, that is, the beginning moments of degradation, which are 10,000min, 10,710 min and 3,075 min, respectively. However, the beginning moments of degradation may be unfaithful because of the randomness of Kurtosis factor, especially when the impulse in Kurtosis factor is very weak, such as in the test 3 failure bearing. (4) The same feature for different bearings varies greatly because of the individual differences, even though at the same period. For examples, RMS of test 1 failure bearing is clearly less than that of tests 2 and 3 failure bearings at the normal period. (5) The overall trend of the original features is fuzzy, while their every point has the strong randomness. Therefore, none of the original features are suitable to assess the performance degradation over the whole lifetime.

**Figure 8.**
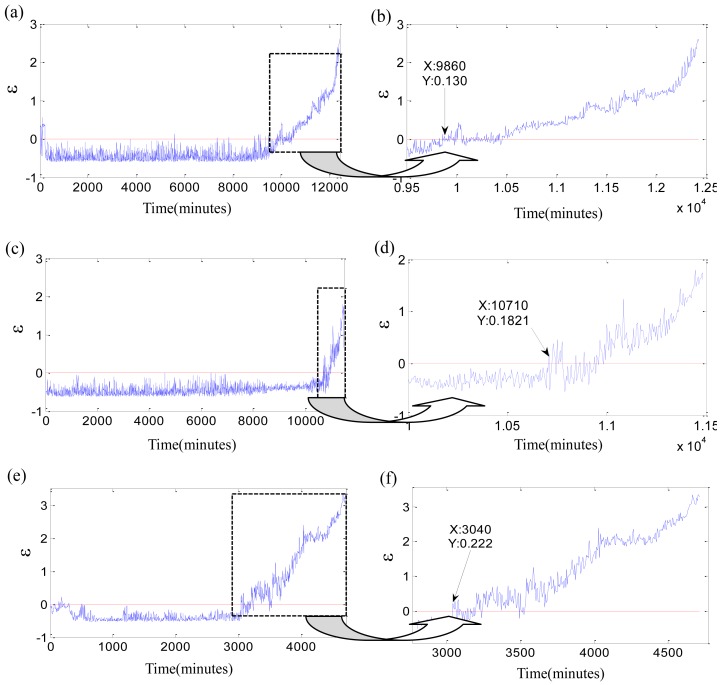
Results of SVDD for tests 1–3 failure bearing: (**a**) *ε* of test 1 failure bearing during the whole life; (**b**) local enlargement of *ε* for test 1 failure bearing; (**c**) *ε* of test 2 failure bearing during the whole life; (**d**) local enlargement of *ε* for test 2 failure bearing; (**e**) *ε* of test 3 failure bearing during the whole life; (**f**) local enlargement of *ε* for test 3 failure bearing.

Secondly, the vector *x_i_* is constructed by the four original features and imported into SVDD to compute the monitoring coefficient *ε*. Figure 8 provides the parameter *ε* and its local enlargement of three bearings.

**Figure 9.**
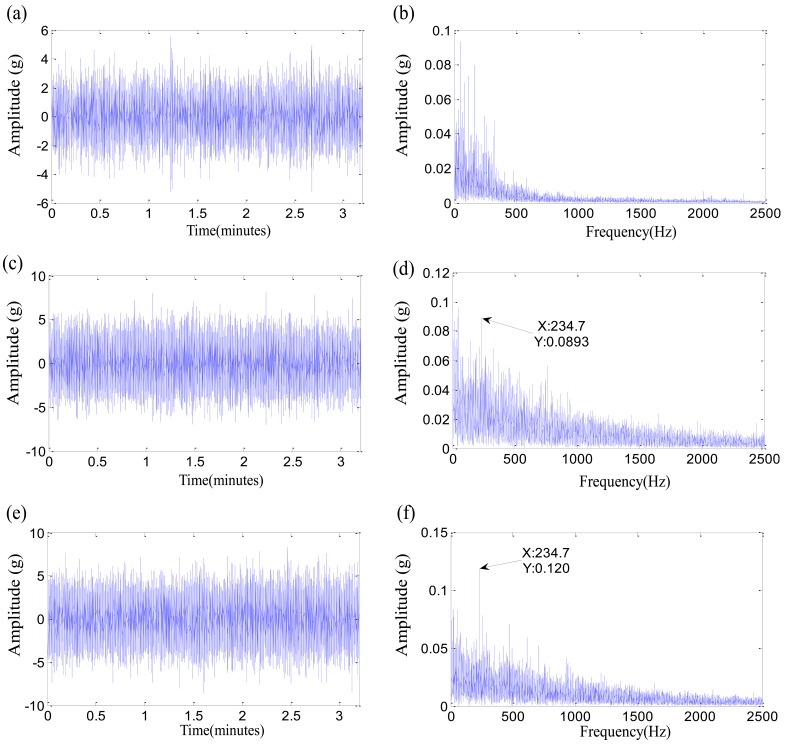

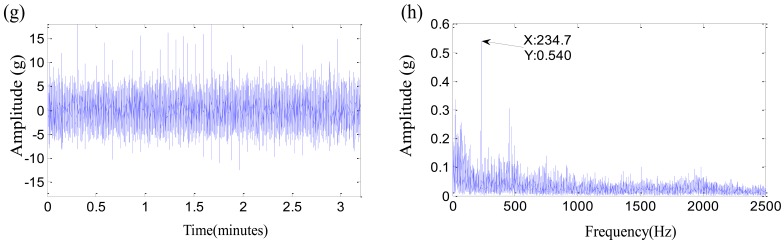
The Time-domain waveforms and Hilbert spectrums of test 1 failure bearing: (**a**) Time-domain waveform on 4,000 min; (**b**) Hilbert spectrum on 4,000 min; (**c**) Time-domain waveform on 9,860 min; (**d**) Hilbert spectrum on 9,860 min; (**e**) Time-domain waveform on 10,000 min; (**f**) Hilbert spectrum on 10,000 min; (**g**) Time-domain waveform on 12,250 min; (**h**) Hilbert spectrum on 12,250 min.

**Figure 10.**
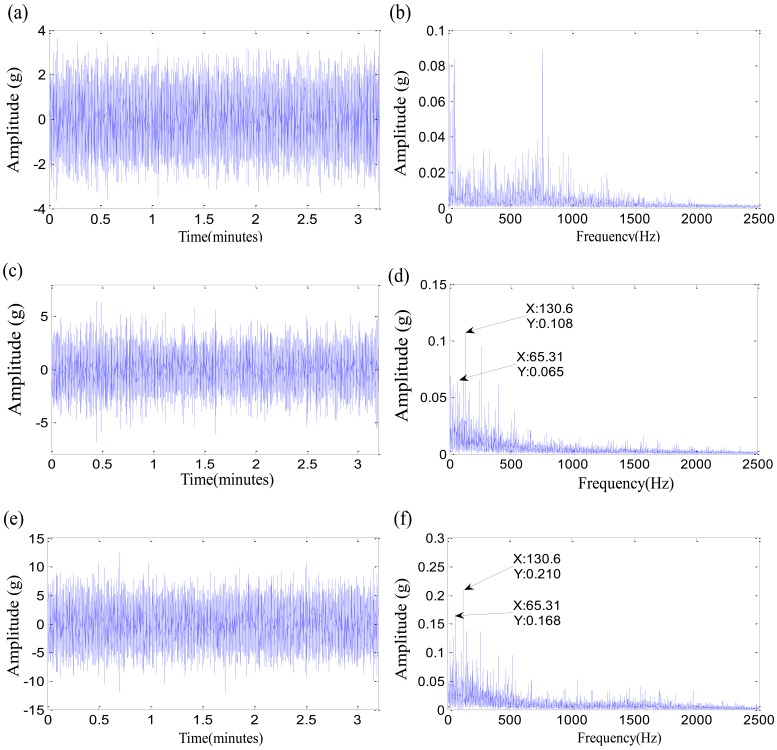
The Time-domain waveforms and Hilbert spectrums of test 2 failure bearing: (**a**) Time-domain waveform on 4,000 min; (**b**) Hilbert spectrum on 4,000 min; (**c**) Time-domain waveform on 10,710 min; (**d**) Hilbert spectrum on 10,710 min; (**e**) Time-domain waveform on 11,400 min; (**f**) Hilbert spectrum on 11,400 min.

**Figure 11.**
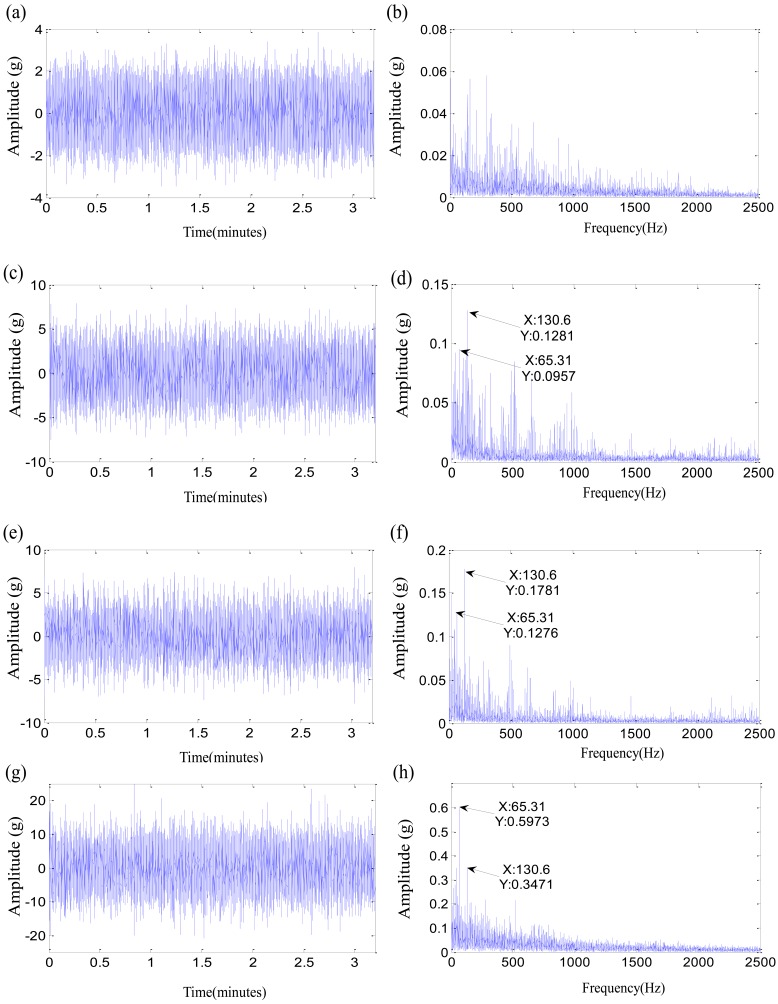
The Time-domain waveforms and Hilbert spectrums of test 3 failure bearing: (**a**) Time-domain waveform on 1,000 min; (**b**) Hilbert spectrum on 1,000 min; (**c**) Time-domain waveform on 3,040 min; (**d**) Hilbert spectrum on 3,040 min; (**e**) Time-domain waveform on 3,075 min; (**f**) Hilbert spectrum on 3,075 min; (**g**) Time-domain waveform on 4,685 min; (**h**) Hilbert spectrum on 4,685 min.

The monitoring coefficient *ε* is a comprehensive index which is sensitive to the initial defect and steadily increases with the damage development. The monitoring coefficient *ε* is utilized to find the initial defect time. The degradation beginning threshold is defined as *ε* > 0. The samples with *ε* > 0 are outside of the sphere acquired by training the normal samples. The two assumptions of Equation (18) are necessary in the processes of the initial defect time determined by the threshold *ε* > 0. They assure that the initial defect time is free from the influence of the vibration randomness, so the moments of 0 ∼ 220 min of test 1 failure bearing are not the degradation beginning time though *ε_i_* > 0, because of the bearing in the run-in period. Likewise, the moments of 5,585 min, 7,340 min, 7,830 min are not the initial defect time because they are the exceptional points. Their *ε_i_* > 0 but their neighbor points are all *ε_i_* < 0. The similar situations appear in the tests 2 and 3 failure bearings. The degradation beginning times of three bearings are determined as 9,860 min, 10,710 min and 3,040 min with *ε_i_* > 0. The new initial defect moments are 40 min, 0 min and 35 min earlier than that determined by Kurtosis factor. Which initial defect time is more accurate? The contrast experiments shown in Figures 9–11 are carried out to verify them.

The left subgraphs are the time domain waveforms at different moments, and the right subgraghs are their corresponding Hilbert spectra. The moment of 4,000 min is the normal stage for the test 1 failure bearing, and the fault characteristic frequencies are not seen in Figure 9(b). A characteristic frequency of 234.7 Hz close to the inner-race defect frequency appears in Figure 9(d), and gradually increases along with the damage development in Figure 9(f,h). Therefore, the moment of 9,860 min could be more suitable as the initial defect time for the test 1 failure bearing. The two initial defect times of test 2 failure bearing are equivalent. The rolling element defect frequency and its second harmonic appear at 10,710 min in Figure 10(d). The contrast experiment of test 3 failure bearing provides the same results as that of test 1 failure bearing. The time determined by *ε_i_* > 0, 3,040 min, is more suitable as the degradation beginning time. However, the monitoring coefficient *ε* is not the ideal indicator. First of all, the parameter *ε* is oscillating, while the actual damages are irreversible. Then the monitoring coefficient *ε* increases slowly with time, which is not able to reflect the accelerated relationship between the damage development and the running time perfectly. That could be as a result of the fuzziness of the damage quantitative. SVDD might need an improvement to deal with the fuzzy damage development.

**Figure 12.**
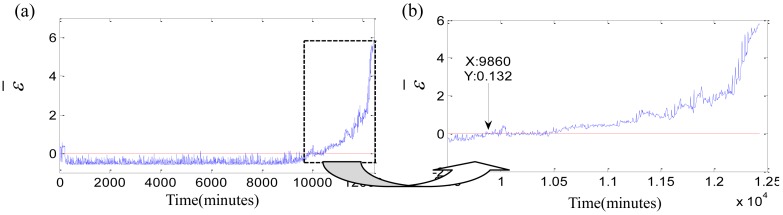

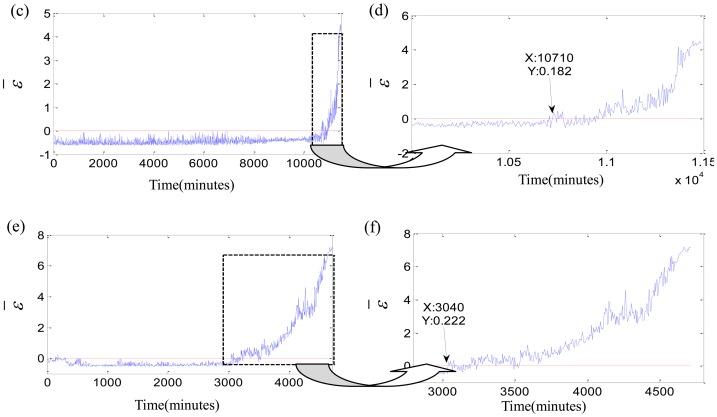
Results of FSVDD for tests 1–3 failure bearing: (**a**) *ε̄* of test 1 failure bearing during the whole life; (**b**) local enlargement of *ε̄* for test 1 failure bearing; (**c**) *ε̄* of test 2 failure bearing during the whole life; (**d**) local enlargement of *ε̄* for test 2 failure bearing; (**e**) *ε̄* of test 3 failure bearing during the whole life; (**f**) local enlargement of *ε̄* for test 3 failure bearing.

**Figure 13.**
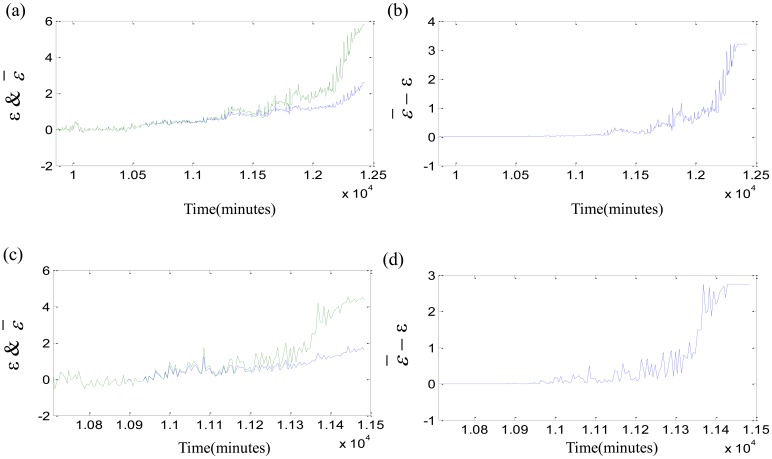

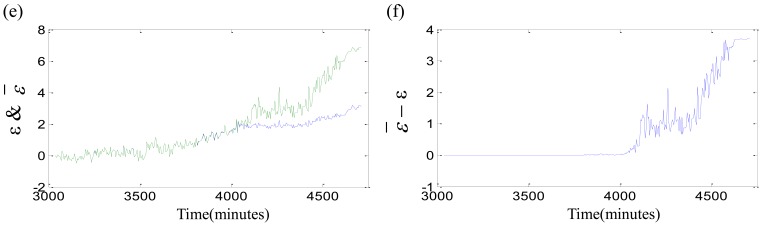
Comparisons between *ε* and *ε̄*: (**a**) *ε* & *ε̄* of test 1 failure bearing; (**b**) *ε̄*− *ε* of test 1 failure bearing; (**c**) *ε* & *ε̄* of test 2 failure bearing; (**d**) *ε̄*− *ε* of test 2 failure bearing; (**e**) *ε* & *ε̄* of test 3 failure bearing; (**f**) *ε̄*− *ε* of test 3 failure bearing.

Thirdly, the fuzzy membership degree *s_i_* is computed by Equation (17). The fuzzy-monitoring coefficients *ε̄* of three bearings are given by FSVDD. The parameter *ε̄* and its local enlargement are described in Figure 12. The fuzzy-monitoring coefficient *ε̄* is an improvement of the monitoring coefficient *ε*, and adds the function of defuzzification. The parameters *ε̄* and *ε* have a certain degree as well as lots of differences. The comparisons between *ε* and *ε̄* are carried out as shown in Figure 13. The parameter *ε* is blue while the parameter *ε̄* is green. They are same at the beginning of degradation, and much different with the damage development. The overall trend of *ε̄* − *ε* is accelerated, but the acceleration is not strict and not able to be proved by the second derivative. The fuzzy-monitoring coefficient *ε̄* has a similar accelerated trend, which could agree with the relationship of the damage development and running time. On the other hand, the D-value of the neighbor *ε̄_i_* gets bigger as the damage increases. The increasing D-value means that the damage severity of the neighbor moments is differentiated easily. However, the fuzzy-monitoring coefficient *ε̄* is still oscillating and not consistent with the irreversible damage development.

**Figure 14.**
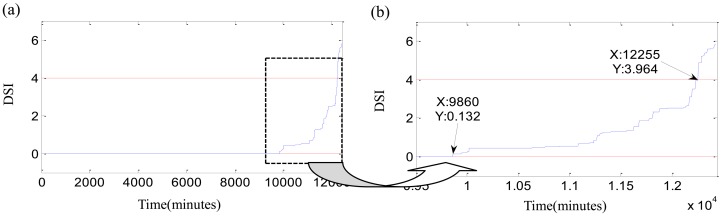

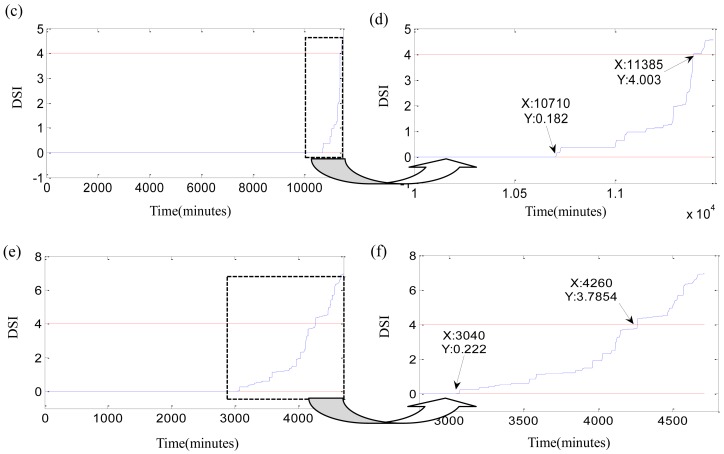
Results of degradation assessment for tests 1–3 failure bearing: (**a**) DSI of test 1 failure bearing during the whole life; (**b**) local enlargement of DSI for test 1 failure bearing; (**c**) DSI of test 2 failure bearing during the whole life; (**d**) local enlargement of DSI for test 2 failure bearing; (**e**) DSI of test 3 failure bearing during the whole life; (**f**) local enlargement of DSI for test 3 failure bearing.

Finally, a new index, DSI, is calculated as Equation (18), and Figure 14 shows the DSI and its local enlargement. The wild points of fuzzy-monitoring coefficient *ε̄* should be rejected before the computation of DSI. The parameter *ε̄* in Figure 12 contains lots of exceptional points. The elimination of wild points assures that DSI does not suffer from vibration randomness. DSI inherits all advantages of the fuzzy-monitoring coefficient *ε̄*, and overcomes its shortcomings. The monotonic index, DSI, reflects the increase of the bearing damages with running time perfectly. Sometimes DSI is jumping which means the occurrence of spalling or the emergence of new damages. On the other hand, the placid DSI implies that the running state of bearing is balanceable and no new damage occurs. The degradation beginning threshold is defined as DSI > 0 according to the monitoring coefficient *ε*. The initial defect occurs at the degradation beginning moment. And the confirmation of the failure threshold should consider the running state of the bearing and the synthetic performance of the whole rotary machine. In this study, the failure threshold is delimited as DSI ≤ 4 The failure moments of three bearings are 12,260 min, 11,385 min and 4,265 min, as shown in Figure 14.

### 4.3. Outer-Race Defect and Inner-Race Defect Simulation

In the run-to-failure tests, there are only two faults types, the inner-race defect and the rolling element defect. Then the outer-race defect and inner-race defect are simulated by increasing the impulse amplitude of the mathematical model to verify again the effectiveness of DSI.

The rolling bearing transfers the main load through elements in rolling contract than in sliding contract [37]. When the defect appears, the characteristic frequencies are seen easily. McFadden developed a model to simulate the vibrations of single point defect [38]. The model contains the effects of rolling element bearing geometry, shaft speed, load distribution, transfer function, and the exponential decay vibration. Then it is extended to describe the vibrations of multiple point defects [39]. The above models provide the cyclical shock, and have been successfully utilized to describe faults in rolling element bearings [40–43]:

(23) $$x(t)=\sum_{i=1}^{M}A_{i}\cdot s(t-iT-\tau_{i})+n(t)$$

where *T* is the period of impulse, *s*(*t*) is the vibration waveform and *n*(*t*) is external noise, *A_i_* is the amplitude modulator to simulate the possible modulated situation, *τ_i_* is the random fluctuation around average period *T*.

Simplifying the vibration waveform *s*(*t*) to an exponential damping cosine signal:

(24) $$s(t)=e^{-Bt}\cdot \cos(2\pi f_{n}t+\varphi_{w})$$

where *B* is the an appropriate value to simulate the attenuation of oscillation waveforms, *f_n_* is the natural frequency related to bearing or system. The amplitude modulator, *A_i_*, is simplified as a cosine signal:

(25) $$A_{i}=A_{0}\cdot \cos(2\pi f_{m}t+\varphi_{A})$$

where *A_0_* is the resonance intensity, *f_m_* = 1/*Q* is the shaft speed for inner-race fault and the cage speed for rolling element fault, and *Q* is the modulated period.

**Figure 15.**
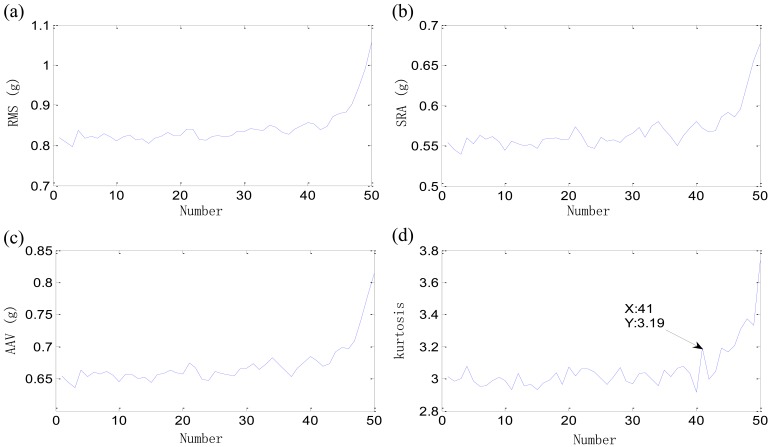

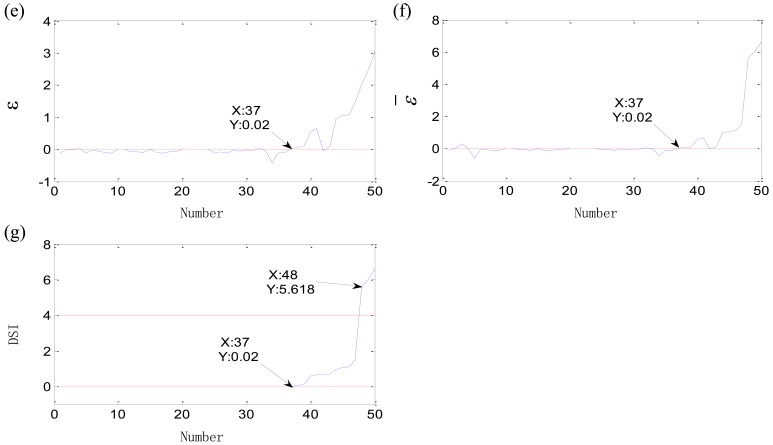
Original features and comprehensive indexes of outer-race defect: (**a**) RMS; (**b**) SRA; (**c**) AAV; (**d**) Kurtosis factor; (**e**) *ε*; (**f**) *ε̄*; (**g**) DSI.

Let *f_m_* = 0 Hz, T = 1/*f_i_* = 0.061 s to simulate the outer-race defect and let *f_m_* = *f_r_* = 25 Hz, T = 1/*f_i_* = 0.042 s to simulate the inner-race defect. The natural frequency *f_n_* = 2,000 Hz. Fifty samples are simulated for each defect. Each simulation sample contains 32,768 points. The initial defects of the outer-race defect and inner-race defect are at about the 40th sample. The original features and comprehensive indexes of outer-race defect are shown in Figure 15. The initial defect is considered as the 41st sample by Kurtosis factor. The randomness of original features is big due to the strong noise in the time-domain waveform. The new initial defect location is determined at the 37th sample by the threshold *ε* > 0 and earlier than that determined by Kurtosis factor. The failure of outer-race defect appears at the 48th sample by the threshold DSI ≤ 4. The similar rule is seen for the inner-race defect. Kurtosis factor determines the initial defect at 41st sample, while the fuzzy-monitoring coefficient confirms the time at 39th sample. The final failure of inner-race defect is confirmed at 48th sample by DSI. Besides, the parameters *ε* and *ε̄* both have many wild points as result of the randomness of original features. DSI is an excellent index which effectively reflects the irreversible development of the bearing defect.

In a word, DSI is an excellent degradation assessment index for rolling bearings and has at least the following advantages: (1) DSI is sensitive to the initial defect and grows stably with the development of faults. The stability features, such as RMS, SRA, AAV, reflect the damage development, but it is hard to find the initial defect. The sensitivity feature as Kurtosis Factor is the opposite. DSI is an excellent indicator which is increasing with the damage development and sensitive to the initial defect. Moreover, the run-to-failure experiment and simulation both verify that DSI determines the initial defect earlier. (2) DSI with the defuzzification ability reflects the accelerating relation between the damage development and running time. This advantage of DSI comes from the fuzzy-monitoring coefficient *ε̄*. The parameter *ε̄* describes the accelerated development of bearing damage as a result of the fuzzy membership degree *s_i_*. However the acceleration is not strict and unable to be verified by the second derivative. (3) The monotonic DSI reflects the irreversible development of the bearing defects. The damage development is irreversibly increasing after the initial defect occurs. The monotonic indicator is more effective to reflect the degradation process.

The type and width parameter of the kernel function affect the parameters *ε* and *ε̄*, even DSI, as described in reference [24]. In this study, Gaussian Raial Basis Function (GRBF) function was adopted due to its good property and universality. The width parameter was calculated carefully by the ex-ante fault diagnosis experiments. At beginning, we selected some normal samples and several of fault samples from the vibration of each failure bearing. The range of width parameters was estimated by the rules of thumb [44]. In the range, the width parameter was sampled with the same interval. The classification error with each sample of the width parameter was calculated by the classifier. The best value range was decided by the minimum error. The width parameter was selected as width = 2 which is in the overlay region of the best ranges for the three failure bearings. The selection of type and width parameter is worth further study for the better degradation assessment.

## 5. Conclusions

This paper presents a study of degradation assessment based on FSVDD and the running time. SVDD constructs the monitoring coefficient *ε* with the advantages of sensitivity to the initial defect and stable growth as the damages develop, but SVDD could not deal well with fuzzy damage severity degree. FSVDD introduces the fuzzy membership degree *s_i_* to SVDD and provides a better assessment index *ε̄*. It could describe the accelerated relation between the development of the damages and running time. However, the index *ε̄* with a oscillating trend disagrees with the actual damage development. Finally a monotonic degradation assessment index, DSI, is constructed. DSI inherits all the advantages of *ε̄* and reflects the irreversible growth of the bearing damages with running time well. Bearing run-to-failure tests and simulation experiments were carried out to validate the proposed method. The results show that DSI reflects the running state of rolling bearing in real time and effectively guarantees the operation reliability of bearings.

Although the analysis results in this study are acceptable, more tests are necessary for further analysis. Meanwhile, other mathematical methods, such as Principal Component Analysis (PCA), can be used to construct some new indexes. PCA uses an orthogonal transformation to convert a set of possibly correlated variables into linearly uncorrelated variables. The transformation is defined in such a way that the first principal component has the largest possible variance, and each succeeding component in turn has the highest possible variance under the constraint which is orthogonal to the preceding components. If the statistical features are input into PCA, many separate composite characteristics are obtained. Some of the composite characteristics may be excellent assessment indexes.

In addition, DSI could be applied to the performance degradation assessment of other key machine components, such as gears, shafts and ball screws, because their dynamic performance is similar to that of bearings. The remaining life prediction could be carried out by the combination of DSI and the regression methods such as SVM, ANN *etc.* DSI is used to determine the time of the initial defect and the finial failure, and the regression methods predict the remaining life by inputting one or many fine indexes like DSI. It could take all advantages of DSI and the regression methods for accurate life prediction.

## Figures and Tables

**Figure 16.**
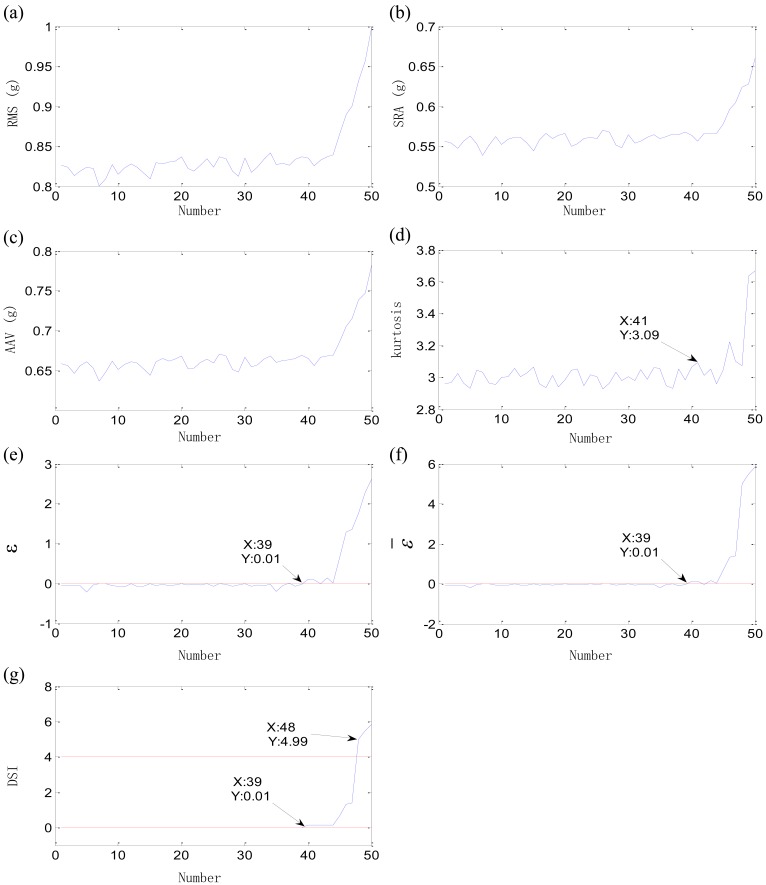
Original features and comprehensive indexes of inner-race defect: (**a**) RMS; (**b**) SRA; (**c**) AAV; (**d**) Kurtosis factor; (**e**) *ε*; (**f**) *ε̄*; (**g**) DSI.

**Table 1.** Parameters of test bearings and steady bearings.

**Bearing Type**	**Inner Diameter (mm)**	**Outer Diameter (mm)**	**Roller Diameter (mm)**	**Roller Number**	**Calculating Coefficient**		**Basic Dynamic Load (kN)**
					e	Y	
30311	55	120	16.25	16	0.35	1.7	152
N312	60	130	19.1	16	—	—	212

**Table 2.** Bearing characteristic frequencies.

*f_r_* **(Hz)**	*f_c_* **(Hz)**	*f_o_* **(Hz)**	*f_i_* **(Hz)**	*f_e_* **(Hz)**
25	10.2393	163.8288	236.1712	65.1061
